# Longitudinal Relationships Between Reading Abilities, Phonological Awareness, Language Abilities and Executive Functions: Comparison of Low Risk Preterm and Full-Term Children

**DOI:** 10.3389/fpsyg.2020.00468

**Published:** 2020-03-17

**Authors:** Miguel Pérez-Pereira, Zeltia Martínez-López, Lorena Maneiro

**Affiliations:** ^1^Department of Developmental and Educational Psychology, University of Santiago de Compostela, Santiago de Compostela, Spain; ^2^Department of Clinical Psychology and Psychobiology, University of Santiago de Compostela, Santiago de Compostela, Spain

**Keywords:** reading abilities, preterm children, executive functions, language developement, phonological awareness (PA), path analysis, Predictive factors, low risk

## Abstract

Different investigations have found that very or extremely (high risk) preterm children show global or specific difficulties in phonological awareness (PA) and reading abilities. Do low risk preterm children, however, exhibit this pattern? Phonological awareness has been considered an important predictor of literacy. Certain executive functions (EFs), and language abilities in turn, have been found to predict PA. The aims of this study are (1) to compare reading abilities of low risk preterm children of different gestational age (GA) groups to those of full-term children, (2) to compare the performance of low risk preterm children of different GA groups to that of full-term children in different EFs, language measures and PA, and (3) to perform a path analysis in order to test a longitudinal model of the relationships between EFs, language abilities, PA and reading. The participants were 108 low risk 4- year-old preterm children, classified into 3 groups of different GAs, and 34 full-term children. The children’s EFs (rapid automatized naming task (RAN), working memory, and inhibition) were assessed at 4 and 5 years of age. Vocabulary comprehension, morphosyntactic production and grammar reception were assessed when the children were 5 years of age, as well as phonemic awareness and syllabic awareness. Finally, reading abilities were assessed when the children were 9 years old. No significant difference between gestational age groups was found on any of the measures taken on EFs, language abilities, phonological awareness, or reading abilities. The path analysis model demonstrates the direct effect of working memory, certain linguistic skills governed by rules (grammar, morphology) and phonological awareness on reading abilities at 9 years of age. The model also shows the mediating role that PA has on the relationship between EFs and language abilities with reading abilities.

## Introduction

The main aim of this study is to analyze those factors which predict reading ability in a group of healthy preterm children and a group of full-term children through a path analysis as well as the mediating effect of phonological awareness (PA) in those relationships. We also intend to compare reading abilities and PA among four groups of children with different gestational ages (three of them healthy preterm and one full-term), as well as their performance in different executive functions (EFs) and language measures.

### Predictors of Reading Ability

Reading abilities have aroused great interest in the scientific community, and particularly the identification of those factors which predict the acquisition of reading competence. This identification can have important consequences for the promotion of reading ability, which is of crucial importance for children’s educational development.

Phonological awareness and rapid automatized naming (RAN) were identified as important predictors of reading abilities in typically developing children (TD) (Swanson et al., 2003; Melby-Lervag et al., 2012; Norton and Wolf, 2012). PA is related with conscious access to the phonological structure and components of words. RAN is the ability to quickly name aloud series of familiar letters, numbers, colors, or objects, which is related to speed processing, sustained attention and response inhibition, and lexical retrieval.

Evidence exists that PA and RAN uniquely contribute to different aspects of reading, and that the combination of deficits in both of them (*double deficit hypothesis*) produces more pervasive and severe reading impairments than single deficits in either RAN or PA (Wolf and Bowers, 1999; Schatschneider et al., 2002; Kirby et al., 2003; Papadopoulos et al., 2009; Vander Stappen and Reybroeck, 2018). Phonological awareness, letter-sound knowledge and alphanumeric RAN were all found to be strong independent predictors of reading development in two longitudinal studies (Caravolas et al., 2013; Clayton et al., 2019). Schatschneider et al. (2002), however, have pointed out the difficulty in establishing the relative impact of RAN deficits on reading ability independent of deficits in PA. In contrast to the evidence accumulated, Swanson et al. (2003) in a meta-analysis have suggested that the importance of RAN and PA measures in accounting for reading ability has been overstated.

In addition to PA and RAN, other studies have indicated that other precursors may have an impact on reading. Oral language development was found to be a strong predictor of reading ability in TD as well, and of reading comprehension in particular. A great number of studies have indicated the similarities between children with specific language impairment (SLI) and dyslexia (Bishop and Snowling, 2004), and have also indicated that children with SLI or language delay have a significantly higher probability than TD of showing subsequent reading impairments (Conti-Ramsden et al., 2001; Catts et al., 2002; Rescorla, 2002; Joye et al., 2019). Children diagnosed with dyslexia may have not only difficulties in phonological processing, but also in semantics, syntax and discourse (Bishop and Snowling, 2004; van Rijthoven et al., 2018). Deficits in phonological skills were found to be strong predictors of reading difficulties (Russell et al., 2018), although other linguistic abilities were also found to predict reading difficulties. Among them, expressive vocabulary, receptive vocabulary, and syntax have been mentioned as predictors of reading comprehension (Muter et al., 2004; Swanson et al., 2008; Lervag and Aukrust, 2010; Kieffer, 2012; Durand et al., 2013).

Similar to SLI children, children with reading impairment also show problems in working memory and other executive functions (Brosnan et al., 2002; Reiter et al., 2005). These affect phonological processing and phonological awareness, which, in turn, are strongly involved in the reading process. In studies carried out with TD children and children with dyslexia, verbal working memory and complex visuospatial memory were predictors of reading comprehension (Smith-Spark et al., 2003; Soriano and Miranda, 2010; Menghini et al., 2011; López-Escribano et al., 2013; Wang and Gathercole, 2013). Arnell et al. (2009) concluded that working memory encoding underlies part of the relationship between RAN and reading ability.

In a finding that is especially relevant for the aims of our study, Knoop-van Campen et al. (2018), found a mediation effect of PA on the relation between working memory and word reading efficiency in children with dyslexia: working memory affected word reading through PA. It is also theoretically sensible that phonological awareness mediates in the influence of language development on reading.

### Reading and Associated Abilities in Preterm Children

Most studies on reading abilities in preterm children (PT) were carried out with very preterm (VPT) or extremely preterm (EPT) children (gestational age < 32 weeks), and the results indicate that school-aged PT children obtain significantly lower results than full-term children (FT) in decoding abilities (Anderson et al., 2003; Johnson et al., 2012, 2016; Taylor et al., 2016; Alanko et al., 2017; Guarini et al., 2019), reading comprehension (Lee et al., 2011) or in both decoding abilities and reading comprehension (Pritchard et al., 2009, 2014; Johnson et al., 2011; Leijon et al., 2016, 2018). Similar results were found in two meta-analyses (Aarnoudse-Moens et al., 2009; Kovachy et al., 2015).

If the predictive variables of reading indicated before (PA, RAN, language, and EFs) are delayed in PT children, it is logical to think that PT children will show reading problems, given their role as precursors of reading abilities. The studies on these abilities in PT children have been mostly carried out with EPT or VPT children, as well. The few studies carried out with PT children of a wider GA range shed doubts on the idea of a general deficit in language development or EFs of PT children. In this regard, it is important to remember that EPT and VPT children represent only 20% of the total number of the PT population, and, therefore, there is a risk of making overgeneralizations from the investigation of VPT and EPT children to the general population of PT children.

In the same way, different studies with VPT or EPT children pointed to the existence of a deficit in PA and RAN in this population at 8 years of age (Alanko et al., 2017; Leijon et al., 2018).

Language delay has been commonly reported in EPT and VPT children (see Barre et al., 2011 for a meta-analysis). In contrast, healthy PT children with a wider GA range seem to progress in language similarly to TD in two studies using the same sample as reported here (Pérez-Pereira et al., 2014; Pérez-Pereira and Cruz, 2018).

Abundant investigation supports the conclusion that EPT and VPT children have deficits in EFs, such as working memory, attention, inhibition or flexibility, as compared to FT children (see van Houdt et al., 2019 for a meta-analysis). However, a study carried out with a sample of healthy PT of wider GA range (mean GA = 32.6, SD = 2.5) (the same sample as in this study) did not observe significant differences with FT children in working memory, inhibitory control and sustained attention (Pérez-Pereira et al., 2019).

The studies on the predictors of reading ability in PT children are scarce. Wocadlo and Rieger (2007) found that low performance in RAN increases the probability of difficulties in academic skills, including reading, in VPT children.

Guarini and Sansavini (2012) and Guarini et al. (2010) found that language (vocabulary, grammar, and PA) and short-term verbal memory had a predictive role on literacy at the age of 8 years for VPT children.

Anderson et al. (2018) studied the effect of the implementation of a working memory training program (Cogmed) on academic achievement (including word reading, spelling, sentence comprehension and mathematics), as well as on working memory, attention and executive behavior, in a sample of 7-year-old EPT children. No positive effect of the training was observed 24 months later.

Rose et al. (2011) using Structural Equation Modeling (SEM) found negative effects of prematurity on reading fluency (but not on letter-word identification), and these effects were mediated by processing speed and executive functions, working memory in particular. The authors found a cascade effect, in which prematurity negatively influenced processing speed, which then influenced EFs, which in turn influenced academic achievement (including reading). Working memory influenced reading independently of inhibition and shifting.

Borchers et al. (2019) studied the effect of a series of variables (PA, language, executive function, and non-verbal IQ) assessed at 6 years of age on text reading skills measured at 8 years of age in a group VPT children and a control group of FT children. VPT children had lower scores than FT children on all measures. Linear regressions analysis revealed that PA and language abilities predicted reading in both groups (accounting for 19.9 and 25.0% of variance, respectively, *p* < 0.001). Executive function and non-verbal IQ predicted reading only in children born preterm.

The novelty of the present investigation is that a great number of possible explanatory (exogenous) variables of reading abilities are studied in a longitudinal design in order to search for a good fit path analysis model which depicts dependencies among those variables. In addition, the sample differs from most previous studies, since it is composed of a wide range of GA variety (26-36 weeks), and the children did not show serious additional medical conditions, which makes it reasonable to think that it is a low risk sample.

The aims of the study are:

(1) To compare reading abilities among four groups of children with different gestational ages (three of them preterm and one full-term).
(2) To compare the performance of the same four groups in the possible predictive variables of reading abilities: PA, RAN, working memory, and language abilities.
(3) To analyze those factors which predict reading ability through a path analysis.
(4) To assess the potential mediating role of PA on the relationship between RAN, working memory, and language abilities in relation to reading abilities.

The hypotheses of the study are:

(1) Given the characteristics of the sample and the results obtained in previous studies, no significant difference in reading abilities will be found among GA groups.
(2) No significant differences will be found, either, in the independent variables studied among GA groups.
(3) Phonological awareness, RAN, working memory, and morphosyntactic development will have a determinant role on reading abilities, with a mediating role of PA.

## Materials and Methods

### Participants and Procedure

The participants form part of a longitudinal sample of children followed since birth. The children were recruited from four different neonatal units of hospitals in Galicia (Spain) at birth. Parents’ consent was previously obtained, as well as the authorization of the Galician Ethics Committee of Clinical Research.

The initial sample was 151 PT children and 49 FT children. The group of PT children had a mean GA of 32.60 (*SD* = 2.43, range 26–36), and the FT group had a mean GA of 39.84 (*SD* = 1.44, range 37–42). The mean Apgar scores (1 min) of the PT and FT children were similar: PT mean = 7.87, *SD* = 1.43, and FT mean = 8.08, *SD* = 1.25 (*t* (197) = −0.909, *p* > 0.05). The group of PT children did not show additional serious complications. Excluded on discharge from the hospital were those PT children who presented periventricular leukomalacia, intraventricular hemorrhage higher than II, hydrocephalus, genetic malformations, chromosomal syndromes, metabolic syndromes associated with intellectual disability (such as phenylketonuria, galactosemia, or homocystinuria), cerebral palsy or severe motor impairments (as diagnosed up until 9 months of age; no children were excluded between the time of hospital discharge and the following assessment), sensorial impairments, or Apgar scores lower than 6 at 5 min.

Data were collected by trained researchers who visited the children’s homes on three occasions within a 6-year interval. The first wave of data collection was carried out when the children were four years of age, and they were assessed on two EFs (working memory and inhibitory control). The second wave took place when the children were five years of age. They were assessed on RAN, language (morphosyntactic production, comprehension of syntactic structures and vocabulary comprehension), and PA (syllabic awareness and phonemic awareness). The third wave took place at nine years of age, and the children were assessed on RAs (letter name, word reading, pseudoword reading, and text comprehension). At this time, the PT sample consisted of 108 children, and the FT sample of 34 children. Both groups were similar in terms of distribution by gender (χ^2^ (1) = 0.036, *p* > 0.05) and mother’s education (χ^2^ (2) = 1.78 *p* > 0.05). The distribution of the children by GA groups (as shown in Table 1) was as follows: 23.9% between 26 and 31 weeks (very and extremely preterm children), 23.2% between 32 and 33 weeks (moderately preterm children), 28.9% between 34 and 36 weeks (late preterm children), and 23.9% above 36 weeks. The formation of the PT children’s groups was conditioned by the number of children available. We tried to have groups with a similar number of participants.

**TABLE 1:** Descriptive statistics and differences in executive functions, language abilities, phonological awareness, and reading abilities as regards gestational age.

**GA Groups**	**Range**		**Total**	**<32 weeks**	**32–33 weeks**	**34–36 weeks**	**>36 weeks**		

*N* (%)			142	34 (23.9)	33 (23.2)	41 (28.9)	34 (23.9)		
Mean gestational age (SD)	26	42		29.62 (1.46)	32.58 (0.50)	34.81 (0.72)	39.68 (1.52)		

	**Min.**	**Max.**	***M* (*SD*)**	**M (*SD*)**	***M* (*SD*)**	***M* (*SD*)**	***M* (*SD*)**	***F***	**η*p*^2^**
**Executive functions**									
Working memory	13	65	29.45 (9.29)	30.53 (10.60)	27.00 (8.21)	29.90 (10.25)	30.12 (7.43)	0.986	0.02
Inhibitory control	11	55	32.79 (7.37)	31.38 (8.65)	30.31 (7.32)	34.83 (6.48)	34.09 (6.31)	3.152*	0.07
RAN	0	34	3.29 (4.50)	4.06 (6.28)	3.19 (4.05)	3.75 (4.53)	2.09 (2.09)	1.250	0.03
**Language abilities**									
Morphosyntactic production	0	65	41.54 (12.18)	42.19 (12.81)	41.55 (11.29)	39.38 (14.81)	43.59 (81.75)	0.751	0.02
Syntactic structure comprehension	0	72	48.73 (10.09)	51.03 (9.31)	47.13 (9.16)	45.49 (12.12)	52.06 (7.33)	3.623*	0.08
Vocabulary comprehension	21	108	58.94 (11.74)	58.59 (10.99)	57.00 (12.12)	58.27 (11.24)	62.00 (12.59)	1.096	0.02
**Phonological awareness**									
Syllabic awareness	0	28	19.88 (5.02)	16.63 (5.43)	20.06 (4.65)	19.49 (5.97)	20.42 (3.71)	0.246	0.01
Phonemic awareness	0	27	20.43 (5.15)	20.84 (6.09)	20.45 (4.86)	19.74 (5.57)	20.82 (3.92)	0.357	0.01
**Reading abilities**									
Text comprehension	11	16	14.44 (1.14)	14.39 (1.20)	14.57 (1.20)	14.46 (1.07)	14.32 (1.20)	0.178	0.01
Letter names	66.67	250	160.81 (34.95)	160.81 (27.49)	156.54 (40.32)	158.83 (38.43)	168.48 (30.18)	0.457	0.02
Word reading	42.22	190.5	91.23 (29.41)	95.09 (38.23)	89.66 (19.90)	90.24 (31.61)	90.95 (28.30)	0.132	0.01
Pseudoword reading	28.57	102.7	59.14 (16.12)	61.37 (18.81)	56.23 (13.18)	58.16 (16.36)	61.99 (16.78)	0.589	0.06

* < 0.05.

Out of a total of 200 children initially recruited, 142 participated in the present study. The reduction from the original number in the sample was due to experimental drop out. There was no substantial change in the characteristics of the sample, which remain very similar. For instance, the distribution of the children by GA groups in the initial sample was as follows: 24.5% between 26 and 31 weeks, 18.5% between 32 and 33 weeks (moderately preterm children), 32.5% between 34 and 36 weeks (late preterm children), and 24.5% above 36 weeks. The PT and FT groups of the initial sample also had a balanced distribution according to gender and mother’s education (χ2(1) = 0.000, *p* = ≥ 0.05, and χ2(2) = 8.66, *p* > 0.05, respectively). The mean Apgar scores of the initial sample and those of the sample used in this study were very similar (EPT and VPT: 6.90 and 7.24, respectively; MPT: 8.38 and 8.27, respectively; LPT: 8.31 and 8.20, respectively; and FT: 8.08 and 8.18, respectively).

### Measures

#### Demographics

Mothers of the children completed an interview that included socio-demographic information of the family, information on pregnancy, Apgar scores, feeding and health habits, educational level of the parents, etc.

### Executive Functions

The Spanish version of Childhood Executive Functioning Inventory (CHEXI, Thorell and Nyberg, 2008) was used to assess working memory and inhibitory control in daily life in children between 4 and 12 years old. CHEXI is completed by children’s parents and it includes 24-items, 5-point Likert-type format (1 = *absolutely uncertain*, 5 = *very true*). Parents rate how much each assertion is a true description of the behavior of the child (e.g., “Cuando se le pide que haga varias cosas, sólo recuerda la primera o la última”: *When the child is asked to do several things, he/she only remembers the first or the last*). Higher scores indicate greater difficulty in working memory and inhibitory control, and lower scores indicate fewer difficulties in working memory and inhibitory control.

The Rapid Automatized Naming (RAN) subtest of the Spanish version of Clinical Evaluation of Language Fundamentals (CELF-4, Semel et al., 2006) was used to assess naming speed (sustained attention and inhibitory control, and fast processing) in persons between 5 and 21 years of age. Children are asked to name rapidly a set of colors (e.g., “rojo”: *red*), a set of shapes (e.g., “cuadrado”: *square*) and a set of combining shapes and colors (e.g., “triángulo azul”: *blue triangle*) that are each presented in a 6 × 6 matrix. Scores for accuracy in naming (RAN-err) were calculated in the matrix of combining shapes and colors, counting the number of errors committed by the child. The number of errors the child committed evidences the degree to which he/she can sustain self-monitoring (accuracy).

### Language Abilities

The production subscale of the *Test de Sintaxis de Aguado* (TSA, Aguado, 1999) was used to assess the morphosyntactic production in children between 3 and 7 years of age. It consists of 29 items. The first twenty-five items contain two figures each. In each item, the researcher says two sentences (e.g., “La chica mira los perros”: T*he girl looks at the dogs*; “la chica mira al perro”: *the girl looks at the dog*), one after the other, without pointing to any picture. Immediately after speaking, the researcher points to one of the images and he/she waits for the child to repeat the match sentence. Then, the other image is pointed to, so that the child repeats the other sentence. The last four items are items of grammatical closure. The production score is obtained by considering the participant’s use of articles, adverbs, prepositions, passive sentences, negations, reflexive sentences, relative clauses, etc. The child receives one point for each correct sentence given.

The *Comprensión de Estructuras Gramaticales* (CEG, Mendoza et al., 2005) was used to assess the comprehension of syntactic structure in children between 4 and 11 years of age. It consists of 80 sheets that include four pictures each. In each item, the researcher pronounces a sentence (e.g., “El niño que mira a la niña está comiendo”: *The boy who looks at the girl is eating*) and the child points to the image that matches the target sentence. The other three images act as (lexical or grammatical) distractors. The total number of correct answers was used for the analysis.

The *Peabody Test de Vocabulario en Imágenes* (PPVT-III, Dunn et al., 2006) was used to assess vocabulary comprehension in people between 2.5 and 90 years of age. It comprises 192 sheets arranged in order of increasing difficulty. Each sheet includes four pictures. In each item, the researcher pronounces a word (e.g., “vaca”: *cow*) and the child is required to point to the image that best matches that word. The total score is obtained by subtracting the number of errors from the ceiling item.

### Phonological Awareness

The phonological awareness scale of *Del Lenguaje Oral al Escrito-Evaluación* (LOLEVA, Peralbo et al., 2015) was used to assess syllabic and phonemic awareness in children between 3 and 8 years of age. It comprises thirteen tasks: rhyme recognition, initial syllable identification, final syllable identification, initial syllable addition, final syllable addition, initial syllable omission, final syllable omission, initial phoneme identification, final phoneme identification, initial phoneme addition, final phoneme addition, initial phoneme omission and final phoneme omission. Each task includes instructions and two examples that are presented in audiovisual format, except for the omission and addition items. All subscales consist of five items, with the exception of the rhyme recognition subtest, which contain ten. The child receives one point for each correct answer given (out of a possible 70 points).

### Reading Abilities

The *Batería de evaluación de los procesos lectores, revisad*a (PROLEC-R, Cuetos et al., 2007) was used to assess reading capacity. This test can be used with children between 6 and 12 years of age. It consists of nine tasks: identification of letters, same–different, word reading, pseudoword reading, grammatical structures, punctuation, sentence comprehension, text comprehension and listening. In the present study, only the scores of the subscales of identification of letters, word reading, pseudoword reading and text comprehension were used. The identification of letter task consists of a list of 20 letters (e.g., “g”); the word reading task consists of a list of 40 words that vary in length, frequency of use, and the complexity of their syllabic structure (e.g., “peine”: *comb*); the pseudoword reading task consists of a list of 40 invented words (e.g., “pueña”). In these three tasks, the researcher points to the item (letter, word or, pseudoword) and the child reads it out loud. In each task, the child receives a *precision* score, measured as the sum of the correct answers, and a *speed* score, measured as the time taken to complete the task. A combined score (*efficiency*) is calculated by dividing the *precision* score by the *speed* score and multiplying the result by 100. The efficiency score was used for the analyses in this study.

Last, the text comprehension task consists of two narrative and two expositive texts. For each text, the children have to respond to four written questions, 16 total responses (e.g., “¿Para qué sacó varias monedas de la hucha?”: *Why did she take a few coins out of the piggybank?*). The child receives one point for each correct answer given.

In all cases raw scores were used for the analyses, not percentile or scalar scores.

### Data Analysis

Firstly, a set of ANOVAs were carried out in order to analyze differences in EFs, language, PA, and reading abilities as regards GA. Partial eta square was used as the estimator of the magnitude of differences between groups. Secondly, zero-order correlations were computed aimed at the examination of inter-relationships among all the study variables. Finally, the effect of EFs, language and PA on reading, as well as the mediating role of PA in the relation between EFs and language on reading, was examined by means of path analysis, which permits the simultaneous modeling of several related regression relationships. Path analysis was selected because it allows for the examination of more complex models including the analysis of the relationships with a set of observed dependent variables as well as mediation effects. The effect of gender and gestational age was controlled. The model was estimated by the *Maximum likelihood* (ML) method and the following goodness of fit indexes were used for the assessment of the model fit: comparative fit index (CFI), Tucker-Lewis index (TLI), root-mean-square error of approximation (RMSEA), and standardized root mean squared residual (SRMR). According to Hu and Bentler (1999) suggestions, RMSEA and SRMR values lower or equal to 0.06, and TLI and CFI values of 0.95 or higher were considered indicators of excellent model fit. Given that the variables in the model were directly observed and all direct and indirect effects were freely estimated however, the simple mediation path model would be just-identified leading to a perfect model fit. Descriptive analysis and zero-order correlations were conducted on IBM SPSS Statistics 24, whereas path analysis was carried out in MPLUS 7.4 (Muthén and Muthén, 2011).

## Results

### Descriptive Statistics and Differences Between PT and FT Children

Descriptive information and differences on EFs, language, PA, and reading abilities as regards GA are presented in Table 1. In order to delve into the specificities of the development of children born prematurely, preterm children were categorized into three different groups according to their weeks of gestation (i.e., < 32 weeks, 32-33 weeks, and 34-36 weeks), whereas the full-term children correspond to the gestation group of more than 36 weeks. The formation of the PT children’s groups was conditioned by the number of children available. We tried to have groups with a similar number of participants. The results showed significant differences between GA groups on inhibitory control and syntactic structure comprehension. The Tukey’s HDS *post hoc* test evidenced higher scores (which indicate unfavorable performance) in inhibitory control in children born with 34-36 weeks of gestation than those born at 32-33 weeks, as well as higher scores (which indicate favorable results) in syntactic structure comprehension of full-term children as compared with children born at 34-36 weeks. Even then, the results of the ANOVAs revealed a lack of significant differences between preterm and full-term children in the remaining variables, including EFs, language, PA, and reading.

### Zero-Order Correlations Between EFs, Language, PA, and Reading

Zero-order correlations between all the variables of study are presented in Table 2. The corresponding findings indicated a significant positive relation of working memory with inhibitory control. We also found significant negative associations of working memory with morphosyntactic production, syntactic structure comprehension, text comprehension, and letter names, as well as between inhibitory control and syntactic structure comprehension, and pseudoword reading. These results mean that the lower the working memory and the inhibitory control problems, the higher the language and reading scores, and viceversa. A significant negative association was also found between RAN and morphosyntactic production, RAN and vocabulary comprehension, and RAN with phonemic awareness. On the other hand, the results showed significant positive associations of morphosyntactic production with syntactic structure comprehension, syllabic awareness, phonemic awareness, and letter names; significant correlations of syntactic structure comprehension with vocabulary comprehension and PA, both syllabic and phonemic awareness; significant inter-relations of syllabic awareness with phonemic awareness and word reading; and highly significant associations among letter names, word reading and pseudoword reading.

**TABLE 2:** Zero-order correlations between executive functions, language abilities, phonological awareness, and reading abilities.

	**1**	**2**	**3**	**4**	**5**	**6**	**7**	**8**	**9**	**10**	**11**	**12**
1 Working memory	1											
2 Inhibitory control	0.70***	1										
3 RAN	–0.08	–0.07	1									
4 Morphosyntactic production	−0.20*	–0.09	−0.21*	1								
5 Syntactic comprehension	−0.27***	−0.19*	–0.13	0.56***	1							
6 Vocabulary comprehension	–0.01	0.07	−0.17*	0.11	0.38***	1						
7 Syllabic awareness	0.03	–0.05	–0.17	0.40***	0.34***	0.12	1					
8 Phonemic awareness	–0.01	–0.09	−0.25**	0.41***	0.34***	0.05	0.77***	1				
9 Text comprehension	−0.22*	–0.13	0.02	0.14	–0.13	0.02	0.13	0.13	1			
10 Letter names	−0.31**	–0.21	–0.15	0.23*	0.17	0.10	0.11	–0.00	0.14	1		
11 Word reading	–0.19	–0.20	–0.03	0.02	0.01	0.06	0.23*	0.00	0.14	0.44***	1	
12 Pseudoword reading	–0.14	−0.22*	0.00	–0.12	0.09	0.12	–0.13	–0.13	0.07	0.46***	0.52***	1

* < 0.05, ** < 0.01, *** < 0.001.

### Path Analysis Model Including the Relationships Between EFs, Language, PA, and Reading

**FIGURE 1:**
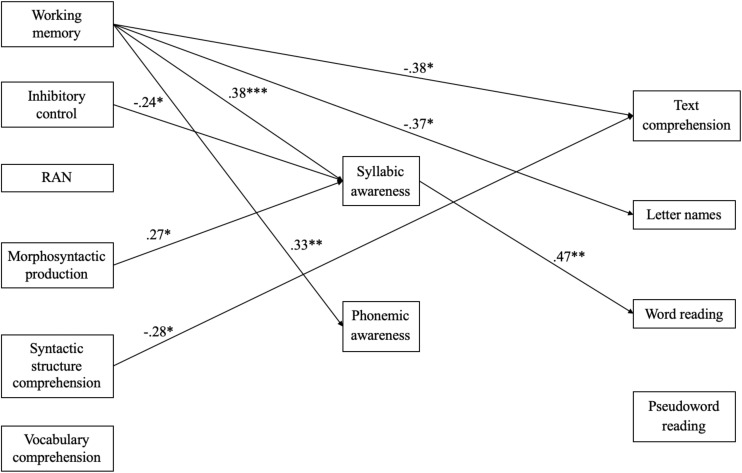
Path analysis model including the relationships between executive functions, language abilities, phonological awareness, and reading abilities. Note. Significant standardized regression coefficients of direct effects between variables are displayed in the path analysis model. ^∗^ < 0.05, ^∗∗^ < 0.01, ^∗∗∗^ < 0.001.

In order to assess both the effect of EFs, language and PA on reading in a longitudinal study, as well as the mediating role of PA on the relationship between EFs and language with reading, a mediating path analysis model was implemented, controlling for the effect of gender and GA (Figure 1). Given the lack of differences found between preterm and full-term children in most of the variables, the whole sample was included in the analysis. The path analysis model evidenced a perfect model fit (CFI = 1.00, TLI = 1.00, RMSEA = 0.00, SRMR = 0.00), because the model was just-identified. The results indicated significant positive direct effects of working memory on syllabic and phonemic awareness as well as significant negative direct effects on text comprehension and letter names. At the same time, inhibitory control showed a significant negative effect on syllabic awareness whereas the latter significantly positively predicted the ability of word reading. Likewise, significant direct effects were found as regards morphosyntactic production on syllabic awareness and syntactic structure comprehension on text comprehension, in a positive and negative way, respectively.

### Indirect Effects of PA on the Relationship Between EFs and Language on Reading

The potential mediating effect of PA, both syllabic and phonemic, on the relationship between EFs and language on reading was also analyzed as part of the path analysis model. The bootstrapping results showed only a single significant indirect effect of syllabic awareness on the relationship between working memory and word reading ability (β = 0.18, *p* < 0.05, 95% CI = 0.058, 0.315).

## Discussion

In relation to the first hypothesis, the results of the ANOVAs analyses confirm, in general terms, our predictions (see Table 1). No significant differences were observed between any of the GA groups in reading abilities analyzed: text comprehension, names of the letters, word reading and pseudoword reading. Partial eta squared values indicate that the magnitude of the differences between the groups was really low. Therefore our results do not agree with previous studies carried out with EPT or VPT children in decoding abilities (Anderson et al., 2003; Johnson et al., 2012, 2016; Taylor et al., 2016; Alanko et al., 2017; Guarini et al., 2019), in reading comprehension (Lee et al., 2011) or in decoding abilities and reading comprehension (Pritchard et al., 2009, 2014; Johnson et al., 2011; Kovachy et al., 2015; Leijon et al., 2016, 2018). It is important to note that the evaluation of reading was done at 9 years of age, and at this age children are supposed to be relatively fluent readers. The differences found with other studies might be related to the age at which reading was assessed. However, the fact that Spanish is a transparent orthography could also affect the results found in our study and might explain, in part, the differences found with other studies which were mostly carried out with non-transparent orthographies. In addition, the fact that the measure we used in certain subtests of the PROLEC-R was efficiency, a mixture of accuracy and time, might also be responsible for the differences found with other studies. In any case, we feel that the main factor which most probably explains the differences found between our results and those of other studies is the low risk characteristic of our sample.

In relation to the second hypothesis, no significant difference was found between the GA groups regarding vocabulary comprehension and morphosyntactic production. The only significant difference (*p* < 0.05) in language was found in grammar structure understanding, with a partial eta squared value of 0.08, which indicates a relatively low magnitude of the differences between the groups. The Tukey’s HDS *post hoc* test indicated that the full-term group had higher scores in syntactic structure comprehension than LPT children born at 34-36 weeks. It is important to note that no significant difference was found between the group of GA < 32 weeks (VPT and EPT) and the full-term group, which evidences that GA was not the factor which could explain this result. Therefore, the results obtained in language reinforce the idea that low risk preterm children progress in language in a way similar to that of TD children (Pérez-Pereira et al., 2014; Pérez-Pereira and Cruz, 2018), contrasting with the results for VPT and EPT children (Barre et al., 2011).

With regards to EFs, no significant difference was found between the GA groups in working memory or RAN. The difference found in inhibitory control (*p* < 0.05) also had an eta squared value of 0.07, which indicates a low effect. Furthermore, we need to consider that the Tukey’s HDS *post hoc* indicates that the difference in inhibitory control was due to the difference between the group of children born with 34-36 weeks of gestation (LPT) and those born at 32-33 weeks (MPT). Again, GA does not seem to be the main cause of this difference. These results contrast with those obtained by other studies carried out with VPT and EPT children (Alanko et al., 2017; Leijon et al., 2018; van Houdt et al., 2019), which showed clear deficits in relation to FT children in several EFs; these results support those found by Pérez-Pereira et al. (2019) with healthy PT children.

There were no significant differences between the four GA groups in any of the PA tests: syllabic awareness and phonemic awareness, in disagreement with the results obtained in other studies carried out with VPT and EPT children (Alanko et al., 2017; Leijon et al., 2018).

To summarize, this study confirms the second hypothesis given that almost no significant differences were found in the independent variables studied among the GA groups. In the two cases where differences were found, GA does not seem to be the cause of the differences.

Finally, the third hypothesis was partially confirmed, since PA and working memory were found to have a strong effect on reading, but, contrary to expectations, RAN was not. In addition, morphosyntactic production had an indirect effect on word reading through syllabic awareness. The path analysis model had a perfect model fit (CFI = 1.00, TLI = 1.00, RMSEA = 0.00, SRMR = 0.00), and was just-identified. The model clearly points out the direct effect that working memory has on text comprehension, syllabic and phonemic awareness and letter names. Therefore, working memory directly affects reading abilities, as other studies have indicated (Rose et al., 2011), reinforcing what has been found with TD children (Smith-Spark et al., 2003; Soriano and Miranda, 2010; Menghini et al., 2011; López-Escribano et al., 2013; Wang and Gathercole, 2013). At the same time working memory had an effect on PA, which, in turn, affected reading. Paradoxically, the direction of the association between working memory and phonological awareness was positive in this case. We would have expected a negative relationship, as in the case of the association between working memory and reading abilities, because lower scores in working memory indicate more favorable results (fewer problems).

The other EFs measured, inhibitory control, also showed a significant effect on syllabic awareness (fewer problems in inhibitory control are associated with better syllabic awareness, and viceversa) whereas the latter significantly positively predicted the ability of word reading. Therefore, a kind of cascade effect was observed, in such a way that EFs affected PA (syllabic awareness), which, in turn affected word reading.

There was a direct effect of syntactic structure comprehension on text comprehension, coinciding with the results found in other studies carried out with PT children (Guarini et al., 2010; Guarini and Sansavini, 2012). Therefore, the ability to understand sentences, which is highly correlated with working memory, logically affects text comprehension, thus confirming that certain linguistic abilities predict reading comprehension in TD (Muter et al., 2004; Swanson et al., 2008; Lervag and Aukrust, 2010; Kieffer, 2012; Durand et al., 2013) and VPT children (Guarini et al., 2010; Guarini and Sansavini, 2012; Borchers et al., 2019). Contrary to expectations, the association between syntactic comprehension and text comprehension was negative. The only explanation we find is that it is a statistical artifact effect, which may also have affected the (positive) association between working memory and phonological awareness. A strong negative association (−0.27, *p* < 0.001) was found between syntactic comprehension and working memory in the zero-order correlations (which is logical since low scores in working memory indicate favorable performance). Paradoxically, the relationship of working memory and syntactic comprehension with text comprehension is negative in the path analysis, when a positive relationship between syntactic comprehension and text comprehension is what one would expect.

The fact that working memory and grammar comprehension are involved in text comprehension is congruent with the dual model of language processing (Ullman, 2001). According to this dual model, syntax, which is rule governed, and EFs are rooted in the same cerebral areas and depend on *procedural* memory processing mechanisms, as opposed to item-based vocabulary learning which depends on *declarative* memory processing. No significant effect of vocabulary comprehension on reading comprehension was found. At the same time, text comprehension and reading ability in general are based in part on the learning of grapheme-phoneme correspondence rules and, therefore, depend on *procedural* model mechanisms.

Morphosyntactic production also affected syllabic awareness. Syllabic awareness, which was highly correlated with phonemic awareness in Zero-order correlations, had a significant mediating effect on word reading.

The mediating effect of PA on the relationship between EFs and language on reading was also analyzed through the path analysis model. The results showed a single significant indirect effect of syllabic awareness on the relationship between working memory and word reading ability. Therefore, working memory seems to have a relevant influence on reading abilities, not only directly but also indirectly through the presence of other factors such as PA.

In general terms, the model is compatible with the third hypothesis, and evidences the effects that working memory, rule governed language (syntax understanding and morphosyntactic production) and PA have on reading (text comprehension, word reading and letter names). However, the effect of RAN was not confirmed. This result is compatible with Arnell et al. (2009) conclusion that working memory encoding underlies part of the relationship between RAN and reading ability; it is also compatible with the suggestion that the relevance of RAN for reading ability has been overstated (Swanson et al., 2003). However, we cannot rule out the explanation that the absence of the effect of RAN on reading ability is related to the measures taken in this study. On one hand, the RAN task used in this study is different from that used in other studies such as Clayton et al. (2019). The accuracy score offered by the task is based on the number of errors (Semel et al., 2006), and we did not take into account the time. On the other hand, the measure we have used in word reading, pseudoword reading and name of letters is not a measure of speed (which would be more sensitive to the effect of speed of processing) but of efficiency. It is quite possible that if time (or a combination of time and accuracy) measures for RAN and speed measures for word reading pseudoword reading and name of letters were taken, the effect of RAM on these decoding abilities would exist.

## Conclusion

Low-risk premature children have no deficiencies in reading ability when compared to FT children, nor do they have them in the predictive factors identified in previous research with VPT or EPT children: oral language, executive functions, phonological awareness.

The path analysis model demonstrates the direct effect of working memory, certain linguistic skills governed by rules (grammar, morphology) and phonological awareness on reading ability. The model also shows the mediating role that PA has on the relationship between EFs and language abilities with reading abilities.

One limitation of the present study is the use of parent report instruments for the assessment of inhibitory control and working memory instead of using experimental tasks.

Probably the type of measures used for certain variables has affected the results found. Future research should explore whether the use of other measures that take more account of time affects the results.

## Data Availability Statement

The datasets generated for this study are available on request to the corresponding author.

## Ethics Statement

The studies involving human participants were reviewed and approved by Comité Ético de Investigación Clínica de Galicia. Written informed consent to participate in this study was provided by the participants’ legal guardian/next of kin.

## Author Contributions

All the authors contributed to the final writing of the manuscript. LM performed the statistical analyses. ZM-L described the Method section. MP-P wrote the introduction and rationale of the study, as well as the discussion and conclusions. The final version is the result of the cooperation among the three co-authors.

## Conflict of Interest

The authors declare that the research was conducted in the absence of any commercial or financial relationships that could be construed as a potential conflict of interest.

## References

[B1] Aarnoudse-Moens C. S. Weisglas-Kuperus N. van Goudoever J. B. Oosterlaan J. (2009). Meta-analysis of neurobehavioral outcomes in very preterm and/or very low birth weight children. *Pediatrics* 124 717–728. 10.1542/peds.2008-2816 19651588

[B2] Aguado G. (1999). *Desarrollo De La Morfosintaxis en el Niño (TSA).* Madrid: CEPE.

[B3] Alanko O. Niemi P. Munck P. Matomaki J. Turunen T. Nurmi J. E. (2017). Reading and math abilities of Finnish school beginners born very preterm or with very low birth weight. *Learn. Individ. Differ.* 54 173–183. 10.1016/j.lindif.2017.01.022

[B4] Anderson P. J. Doyle L. W. Victorian Infant Collaborative Study, (2003). Neurobehavioral outcomes of school-age children born extremely low birth weight or very preterm in the 1990s. *JAMA J. Am. Med. Assoc.* 289 3264–3272. 10.1001/jama.289.24.3264 12824207

[B5] Anderson P. J. Lee K. J. Roberts G. Spencer-Smith M. M. Thompson D. K. Seal M. L. (2018). Long-term academic functioning following cogmed working memory training for children born extremely preterm: a randomized controlled trial. *J. Pediatr.* 202:92. 10.1016/j.jpeds.2018.07.003 30177350

[B6] Arnell K. M. Joanisse M. F. Klein R. M. Busseri M. A. Tannock R. (2009). Decomposing the relation between rapid automatized naming (RAN) and reading ability. *Can. J. Exp. Psychol.* 63 173–184. 10.1037/a0015721 19739900

[B7] Barre N. Morgan A. Doyle L. W. Anderson P. J. (2011). Language abilities in children who were very preterm and/or very low birth weight: a meta-analysis. *J. Pediatr.* 158 766–U100. 10.1016/j.jpeds.2010.10.032 21146182

[B8] Bishop D. V. M. Snowling M. J. (2004). Developmental dyslexia and specific language impairment: same or different? *Psychol. Bull.* 130 858–886. 10.1037/0033-2909.130.6.858 15535741

[B9] Borchers L. R. Bruckert L. Travis K. E. Dodson C. K. Loe I. M. Marchman V. A. (2019). Predicting text reading skills at age 8years in children born preterm and at term. *Early Hum. Dev.* 130 80–86. 10.1016/j.earlhumdev.2019.01.012 30708270 PMC6402954

[B10] Brosnan M. Demetre J. Hamill S. Robson K. Shepherd H. Cody G. (2002). Executive functioning in adults and children with developmental dyslexia. *Neuropsychologia* 40 2144–2155. 10.1016/s0028-3932(02)00046-5 12208010

[B11] Caravolas M. Lervag A. Defior S. Seidlova Malkova G. Hulme C. (2013). Different patterns, but equivalent predictors, of growth in reading in consistent and inconsistent orthographies. *Psychol. Sci.* 24 1398–1407. 10.1177/0956797612473122 23744876

[B12] Catts H. W. Fey M. E. Tomblin J. B. Zhang X. Y. (2002). A longitudinal investigation of reading outcomes in children with language impairments. *J. Speech Lang. Hear. Res.* 45 1142–1157. 10.1044/1092-4388(2002/093) 12546484

[B13] Clayton F. J. West G. Sears C. Hulme C. Lervåg A. (2019). A longitudinal study of early reading development: letter-sound knowledge, phoneme awareness and ran, but not letter-sound integration, predict variations in reading development. *Sci. Stud. Read.* 24 91–107. 10.1080/10888438.2019.1622546

[B14] Conti-Ramsden G. Botting N. Simkin Z. Knox E. (2001). Follow-up of children attending infant language units: outcomes at 11 years of age. *Inter. J. Lang. Commun. Disord.* 36 207–219. 10.1080/13682820121213 11344595

[B15] Cuetos F. Rodríguez B. Ruano E. Arribas D. (2007). *Prolec-R. Batería de Evaluación de los Procesos Lectores, Revisada.* Madrid: TEA.

[B16] Dunn L. M. Dunn D. M. Arribas D. (2006). *PPVT-III, Peabody Test de Vocabulario en Imágenes.* Madrid: TEA.

[B17] Durand V. N. Loe I. M. Yeatman J. D. Feldman H. M. (2013). Effects of early language, speech, and cognition on later reading: a mediation analysis. *Front. Psychol.* 4:586. 10.3389/fpsyg.2013.00586 24027549 PMC3759794

[B18] Guarini A. Bonifacci P. Tobia V. Alessandroni R. Faldella G. Sansavini A. (2019). The profile of very preterm children on academic achievement. A cross-population comparison with children with specific learning disorders. *Res. Dev. Disabili.* 87 54–63. 10.1016/j.ridd.2019.02.001 30772706

[B19] Guarini A. Sansavini A. (2012). Language, executive functions, short-term memory and literacy in preterm children: a longitudinal study. *Riv. Psicolinguist. Appl.* 12 101–115.

[B20] Guarini A. Sansavini A. Fabbri C. Savini S. Alessandroni R. Faldella G. (2010). Long-term effects of preterm birth on language and literacy at eight years. *J. Child Lang.* 37 865–885. 10.1017/s0305000909990109 19698208

[B21] Hu L. T. Bentler P. M. (1999). Cutoff criteria for fit indexes in covariance structure analysis: conventional criteria versus new alternatives. *Struct. Equ. Model.* 6 1–55. 10.1080/10705519909540118

[B22] Johnson S. Marlow N. Wolke D. (2012). Assessing educational outcomes in middle childhood: validation of the teacher academic attainment scale. *Dev. Med. Child Neurol.* 54 544–551. 10.1111/j.1469-8749.2012.04264 22458287

[B23] Johnson S. Strauss V. Gilmore C. Jaekel J. Marlow N. Wolke D. (2016). Learning disabilities among extremely preterm children without neurosensory impairment: comorbidity, neuropsychological profiles and scholastic outcomes. *Early Hum. Dev.* 103 69–75. 10.1016/j.earlhumdev.2016.07.009 27517525

[B24] Johnson S. Wolke D. Hennessy E. Marlow N. (2011). Educational outcomes in extremely preterm children: neuropsychological correlates and predictors of attainment. *Dev. Neuropsychol.* 36 74–95. 10.1080/87565641.2011.540541 21253992

[B25] Joye N. Broc L. Olive T. Dockrell J. (2019). Spelling performance in children with developmental language disorder: a meta-analysis across european languages. *Sci. Stud. Read.* 23 129–160. 10.1080/10888438.2018.1491584

[B26] Kieffer M. J. (2012). Early oral language and later reading development in spanish-speaking english language learners: evidence from a nine-year longitudinal study. *J. Appl. Dev. Psychol.* 33 146–157. 10.1016/j.appdev.2012.02.003

[B27] Kirby J. R. Parrila R. K. Pfeiffer S. L. (2003). Naming speed and phonological awareness as predictors of reading development. *J. Educ. Psychol.* 95 453–464. 10.1037/0022-0663.95.3.453

[B28] Knoop-van Campen C. A. N. Segers E. Verhoeven L. (2018). How phonological awareness mediates the relation between working memory and word reading efficiency in children with dyslexia. *Dyslexia* 24 156–169. 10.1002/dys.1583 29577521 PMC6175128

[B29] Kovachy V. N. Adams J. N. Tamaresis J. S. Feldman H. M. (2015). Reading abilities in school-aged preterm children: a review and meta-analysis. *Dev. Med. Child Neurol.* 57 410–419. 10.1111/dmcn.12652 25516105 PMC4397135

[B30] Lee E. S. Yeatman J. D. Luna B. Feldman H. M. (2011). Specific language and reading skills in school-aged children and adolescents are associated with prematurity after controlling for IQ. *Neuropsychologia* 49 906–913. 10.1016/j.neuropsychologia.2010.12.038 21195100 PMC3078177

[B31] Leijon I. Ingemansson F. Nelson N. Samuelsson S. Wadsby M. (2018). Children with a very low birthweight showed poorer reading skills at eight years of age but caught up in most areas by the age of 10. *Acta Paediatr.* 107 1937–1945. 10.1111/apa.14377 29706015

[B32] Leijon I. Ingemansson F. Nelson N. Wadsby M. Samuelsson S. (2016). Reading deficits in very low birthweight children are associated with vocabulary and attention issues at the age of seven. *Acta Paediatr.* 105 60–68. 10.1111/apa.13094 26098907 PMC4758409

[B33] Lervag A. Aukrust V. G. (2010). Vocabulary knowledge is a critical determinant of the difference in reading comprehension growth between first and second language learners. *J. Child Psychol. Psychiatr.* 51 612–620. 10.1111/j.1469-7610.2009.02185.x 19878367

[B34] López-Escribano C. Elosua de Juan M. R. Gomez-Veiga I. Garcia-Madruga J. A. (2013). A predictive study of reading comprehension in third-grade Spanish students. *Psicothema* 25 199–205. 10.7334/psicothema2012.175 23628534

[B35] Melby-Lervag M. Lyster S. A. H. Hulme C. (2012). Phonological skills and their role in learning to read: a meta-analytic review. *Psychol. Bull.* 138 322–352. 10.1037/a0026744 22250824

[B36] Mendoza E. Carballo G. Muñoz J. Fresneda D. (2005). *CEG: Test de Comprensión de Estructuras Gramaticales.* Madrid: TEA Ediciones.

[B37] Menghini D. Finzi A. Carlesimo G. A. Vicari S. (2011). Working memory impairment in children with developmental dyslexia: is it just a phonological deficity? *Dev. Neuropsychol.* 36 199–213. 10.1080/87565641.2010.549868 21347921

[B38] Muter V. Hulme C. Snowling M. J. Stevenson J. (2004). Phonemes, rimes, vocabulary, and grammatical skills as foundations of early reading development: evidence from a longitudinal study. *Dev. Psychol.* 40 665–681. 10.1037/0012-1649.40.5.665 15355157

[B39] Muthén L. K. Muthén B. O. (2011). *Mplus User’s Guide*, 7th Edn, Los Ángeles: Muthén and Muthén.

[B40] Norton E. S. Wolf M. (2012). Rapid automatized naming (RAN) and reading fluency: implications for understanding and treatment of reading disabilities. *Ann. Rev. Psychol.* 63 427–452. 10.1146/annurev-psych-120710-100431 21838545

[B41] Papadopoulos T. C. Georgiou G. K. Kendeou P. (2009). Investigating the double-deficit hypothesis in greek findings from a longitudinal study. *J. Learn. Disabil.* 42 528–547. 10.1177/0022219409338745 19723979

[B42] Peralbo M. Mayor M. A. Zubiauz B. Risso A. Fernández M. L. Tuñas A. (2015). The loleva oral and written language test: psychometric properties. *Span. J. Psychol.* 18:E18. 10.1017/sjp.2015.15 25850336

[B43] Pérez-Pereira M. Cruz R. (2018). A longitudinal study of vocabulary size and composition in low risk preterm children. *First Lang.* 38 72–94. 10.1177/0142723717730484

[B44] Pérez-Pereira M. Fernandez P. Gomez-Taibo M. Resches M. (2014). Language development of low risk preterm infants up to the age of 30 months. *Early Hum. Dev.* 90 649–656. 10.1016/j.earlhumdev.2014.08.004 25189697

[B45] Pérez-Pereira M. Peralbo M. Veleiro A. (2019). “Prematurity, executive functions and language,” in *Atypical Language Development in Romance Languages*, eds Aguilar-Mediavilla E. Buil-Legaz L. López-Penadés R. Sánchez-Azanza V. Adrover-Roig D. (Amsterdam: John Benjamins Publishing), 37–56. 10.1075/z.223.03per

[B46] Pritchard V. E. Bora S. Austin N. C. Levin K. J. Woodward L. J. (2014). Identifying very preterm children at educational risk using a school readiness framework. *Pediatrics* 134 E825–E832. 10.1542/peds.2013-3865 25113296

[B47] Pritchard V. E. Clark C. A. C. Liberty K. Champion P. R. Wilson K. Woodward L. J. (2009). Early school-based learning difficulties in children born very preterm. *Early Hum. Dev.* 85 215–224. 10.1016/j.earlhumdev.2008.10.004 19022593

[B48] Reiter A. Tucha O. Lange K. W. (2005). Executive functions in children with dyslexia. *Dyslexia* 11 116–131. 10.1002/dys.289 15918370

[B49] Rescorla L. (2002). Language and reading outcomes to age 9 in late-talking toddlers. *J. Speech Lang. Hear. Res.* 45 360–371. 10.1044/1092-4388(2002/028) 12003517

[B50] Rose S. A. Feldman J. F. Jankowski J. J. (2011). Modeling a cascade of effects: the role of speed and executive functioning in preterm/full-term differences in academic achievement. *Dev. Sci.* 14 1161–1175. 10.1111/j.1467-7687.2011.01068.x 21884331

[B51] Russell G. Ukoumunne O. C. Ryder D. Golding J. Norwich B. (2018). Predictors of word-reading ability in 7-year-olds: analysis of data from a UK cohort study. *J. Res. Read.* 41 58–78. 10.1111/1467-9817.12087

[B52] Schatschneider C. Carlson C. D. Francis D. J. Foorman B. R. Fletcher J. M. (2002). Relationship of rapid automatized naming and phonological awareness in early reading development: implications for the double-deficit hypothesis. *J. Learn. Disabil.* 35 245–256. 10.1177/002221940203500306 15493321

[B53] Semel E. Wayne W. Secord A. (2006). *Clinical Evaluation of Language Fundamentals, Spanish Edition (CELF-4). Psychological Corporation.* New York: Harcourt Brace Jovanovich.

[B54] Smith-Spark J. H. Fisk J. E. Fawcett A. J. Nicolson R. I. (2003). Investigating the central executive in adult dyslexics: evidence from phonological and visuospatial working memory performance. *Eur. J. Cogn. Psychol.* 15 567–587. 10.1080/09541440340000024

[B55] Soriano M. Miranda A. (2010). Developmental dyslexia in a transparent orthography: a study of spanish dyslexic children. *Liter. Learn.* 23 95–114. 10.1108/s0735-004x20100000023006 24559885

[B56] Swanson H. L. Rosston K. Gerber M. Solari E. (2008). Influence of oral language and phonological awareness on children’s bilingual reading. *J. Sch. Psychol.* 46 413–429. 10.1016/j.jsp.2007.07.002 19083366

[B57] Swanson H. L. Trainin G. Necoechea D. M. Hammill D. D. (2003). Rapid naming, phonological awareness, and reading: a meta-analysis of the correlation evidence. *Rev. Educ. Res.* 73 407–440. 10.3102/00346543073004407

[B58] Taylor R. Pascoe L. Scratch S. Doyle L. W. Anderson P. Roberts G. (2016). A simple screen performed at school entry can predict academic under-achievement at age seven in children born very preterm. *J. Paediatr. Child Health* 52 759–764. 10.1111/jpc.13186 27189705

[B59] Thorell L. B. Nyberg L. (2008). The childhood executive functioning inventory (CHEXI): a new rating instrument for parents and teachers. *Dev. Neuropsychol.* 33 526–552. 10.1080/87565640802101516 18568903

[B60] Ullman M. T. (2001). A neurocognitive perspective on language: the declarative/procedural model. *Neuroscience* 2 717–725. 11584309 10.1038/35094573

[B61] van Houdt C. A. Oosterlaan J. van Wassenaer-Leemhuis A. G. van Kaam A. H. Aarnoudse-Moens C. S. H. (2019). Executive function deficits in children born preterm or at low birthweight: a meta-analysis. *Dev. Med. Child Neurol.* 61 1015–1024. 10.1111/dmcn.14213 30945271 PMC6850293

[B62] van Rijthoven R. Kleemans T. Segers E. Verhoeven L. (2018). Beyond the phonological deficit: semantics contributes indirectly to decoding efficiency in children with dyslexia. *Dyslexia* 24 309–321. 10.1002/dys.1597 30239065 PMC6282981

[B63] Vander Stappen C. Reybroeck M. V. (2018). Phonological awareness and rapid automatized naming are independent phonological competencies with specific impacts on word reading and spelling: an intervention study. *Front. Psychol.* 9:320. 10.3389/fpsyg.2018.00320 29593618 PMC5859220

[B64] Wang S. M. Gathercole S. E. (2013). Working memory deficits in children with reading difficulties: memory span and dual task coordination. *J. Exp. Child Psychol.* 115 188–197. 10.1016/j.jecp.2012.11.015 23403228

[B65] Wocadlo C. Rieger I. (2007). Phonology, rapid naming and academic achievement in very preterm children at eight years of age. *Early Hum. Dev.* 83 367–377. 10.1016/j.earlhumdev.2006.08.001 16979856

[B66] Wolf M. Bowers P. G. (1999). The double-deficit hypothesis for the developmental dyslexias. *J. Educ. Psychol.* 91 415–438. 10.1037/0022-0663.91.3.415

